# Lower limb alignment becomes more varus and hyperextended from supine to bipedal stance in asymptomatic, osteoarthritic and prosthetic neutral or varus knees

**DOI:** 10.1007/s00167-018-5273-z

**Published:** 2018-11-10

**Authors:** Michael J. C. Brown, Angela H. Deakin, Frederic Picard, Philip E. Riches, Jon V. Clarke

**Affiliations:** 1 Department of Orthopaedics, Golden Jubilee National Hospital, Agamemnon Street, West Dunbartonshire, Clydebank, G81 4DY UK; 2 Department of Biomedical Engineering, University of Strathclyde, Glasgow, G4 0NW Scotalnd UK

**Keywords:** Total knee arthroplasty, Lower limb alignment, Supine, Bipedal stance, Noninvasive, Infrared tracking, Weight-bearing, Tibiofemoral

## Abstract

**Purpose:**

Knee alignment is a fundamental measurement in the assessment, monitoring and surgical management of patients with osteoarthritis. There is a lack of data regarding how static tibiofemoral alignment varies between supine and standing conditions. This study aimed to quantify the relationship between supine and standing lower limb alignment in asymptomatic, osteoarthritic (OA) and prosthetic (TKA) knees.

**Methods:**

A non-invasive position capture system was used to assess knee alignment for 30 asymptomatic controls and 31 patients with OA both before and after TKA. Coronal and sagittal mechanical femorotibial angles were measured supine with the lower limb in extension and in bipedal stance. Changes between conditions were analysed using paired *t*tests. Vector plots of ankle centre displacement relative to the knee centre from supine to standing were produced to allow three-dimensional visualisation.

**Results:**

All groups showed a trend towards varus and extension when going from supine to standing. Mean change for asymptomatic knees was 1.2° more varus (*p* = 0.001) and 3.8° more extended (*p* < 0.001). For OA knees this was 1.1° more varus (*p* = 0.009) and 5.9° more extended (p < 0.001) and TKA knees 1.9° more varus (*p* < 0.001) and 5.6° more extended (*p* < 0.001).

**Conclusion:**

The observed consistent changes in lower limb alignment between supine and standing positions across knee types suggests the soft tissue envelope restraining the knee may have a greater influence on dynamic alignment changes than the underlying bony deformity. This highlights the importance of quantifying soft tissue behaviour when planning, performing and evaluating alignment dependent surgical interventions of the knee. When routinely assessing any type of knee, clinicians should be aware that subtle consistent alignment changes occur under weightbearing conditions and tailor their treatments accordingly.

**Level of evidence:**

II.

## Introduction

Tibiofemoral alignment is a fundamental measurement in the assessment, monitoring and surgical management of patients with knee osteoarthritis (OA) [3]. Alignment in total knee arthroplasty (TKA) is a key consideration when aiming to achieve a well functioning, pain free knee with good longevity. Although exact alignment targets remain a subject of much debate, both on the traditionally accepted limits of ± 3° from neutral on survivorship [1, 4, 14, 19] and the maintenance of pre-operative neutral or varus alignment postoperatively on outcomes [8, 13, 17, 26], the development of modern techniques do allow the surgeon to more accurately achieve the desired alignment [24]. Despite the use of these techniques, a significant minority of patients remain dissatisfied following TKA [2, 21]. This suggests that our knowledge base regarding optimal alignment in TKA may be incomplete with one potential limitation being the potential discrepancy between supine and weight-bearing conditions. The intra-operative measurement of alignment during knee surgery is performed on patients that are supine, with subsequent validation using weight-bearing radiographs. Although there are a number of studies which measure supine and standing alignment they are very much focused on agreement between modalities (attributing variations to measurement differences) so results are not presented in a way to show how actual alignment changes [9, 11, 22, 27, 28]. Therefore it is still difficult intraoperatively for the surgeon to predict what changes may occur in alignment when the patient transfers to a standing position. In spite of extensive research to establish normal alignment values in asymptomatic subjects [7, 11, 15, 22], our understanding of how knee alignment varies when changing from a supine to a standing position remains poor, particularly in patients with OA and in those who have undergone TKA. There is little information available regarding the weight-bearing sagittal mechanical axis of the lower extremity which may be due to the technical difficulty associated with obtaining an adequate full length lateral radiograph [24]. Quantification of any consistent changes in lower limb alignment from supine to a bipedal stance could have clinical implications for surgeons performing alignment dependant procedures on the lower limb. The aim of this study was to quantify the relationship between supine and standing lower limb alignment in asymptomatic, osteoarthritic and prosthetic knees. The hypothesis was that osteoarthritic and prosthetic knees would show different trends in alignment changes to asymptomatic knees.

## Materials and methods

This was a prospective cohort study. The primary outcome with full methodology has already been reported [5] A validated non-invasive infrared position capture system (repeatability 1.6° for coronal and 2.3° for sagittal planes) was used to assess the knee alignment for asymptomatic osteoarthritic (OA) and prosthetic (TKA) knees [6]. A full description of the non-invasive system and method is given in Clarke et al. 2012 [6]. In summary, the hardware was a commercial clinical image free navigation system (OrthoPilot®, BBraun Aesculap, Tuttlingen, Germany) consisting of an optical localizer, active infrared (IR) trackers, and a probe to digitise anatomical landmarks. Mountings for the IR trackers consisted of curved metal base plates and broad straps made from standard-strength elastic webbing (542, E&E Accessories, Kingston upon Thames, UK). Baseplates were firmly applied to thigh, calf and midfoot using the straps and the IR trackers clipped to them. The software was commercial clinical high tibial osteotomy (HTO) software (OrthoPilot® HTO version 1.5 BBraun Aesculap, Tuttlingen, Germany.) The standard registration process required by the software was carried out. Each participant was asked to relax whilst lying supine on an examination couch to ensure that all registration movements were passive. The registration used both kinematics (circumduction of the hip joint, flexion and internal rotation of the knee, flexion of the ankle) and anatomic palpation (epicondyles and malleoli) to determine hip, knee and ankle joint centres. Once registration was complete the software identified the resultant coronal and sagittal MFT angles of the lower limb and these could be tracked as the limb was moved.

### Asymptomatic knees

30 healthy volunteers with asymptomatic knees and no previous surgery were recruited from the university, all giving written informed consent. All kinematic assessments were performed by a single orthopaedic surgeon (JVC) using a validated methodology [6]. In summary infrared trackers were non-invasively attached to the lower limb of each volunteer using material straps and a registration process enabled femoral and tibial motion tracking. Supine coronal and sagittal MFT angles were recorded in maximum extension by supporting the lower limb under the heel without applying any forces. Following the supine assessment the volunteer was asked to assume a normal bipedal stance and the MFT angles were recorded again. After the weight-bearing MFT angles had been recorded the volunteer transferred between supine and standing twice more to repeat the standing registration.

### OA and TKA knees

34 patients with end stage osteoarthritis due to undergo TKA at our institution were approached to take part in the study. Thirty-one patients gave written informed consent to participate. These patients were measured using the same protocol as the asymptomatic knee both preoperatively (OA group) and at 6 weeks postoperatively (TKA group) to give MFT angles. All patients underwent navigated TKA with Columbus® cruciate retaining implants (BBraun Aesculap, Tuttlingen, Germany) as per the standard practice of the two consultants involved. One patient was excluded from the TKA group because of a deep seated infection requiring further operative intervention.

For the asymptomatic knees, healthy volunteers were recruited through the University of Strathclyde. The University of Strathclyde Ethics Committee provided ethical approval for the study (090957). Patients with end stage OA were recruited for the OA and TKA groups following ethical approval from the West of Scotland Research Ethics Committee 2 (07/S0709/27).

### Statistical analysis

Coronal MFT angles were defined as negative for varus and positive for valgus and the sagittal MFT angles as negative for hyperextension and positive for flexion. Statistical analysis was completed using Excel 2007 (Microsoft Corp, Redmond, WA, USA) and SPSS 17.0 (SPSS Inc., Chicago, IL, USA). Data were assessed for normality and paired *t* tests were used to assess changes in alignment between supine and standing positions for each group. A *p* value of < 0.05 was considered significant. To visualise the change in three dimensions, plots of the ankle centre displacement relative to the knee centre were produced in the transverse plane using Matlab (MathWorks Inc, Natick, MA, USA). The displacements were determined as fractions of tibial length rather than absolute distance measurements in order to normalise the displacements. The origin of the vector was the supine position of the ankle centre relative to the knee centre and the end of the vector was the position after weight-bearing. Therefore the starting point was dependent on the initial coronal and sagittal alignment of the subject. To provide a clear representation of the relative supine to standing alignment change, and for comparing groups, the displacements were plotted from a common point of origin regardless of initial alignment. This paper reports the secondary outcome of another study and so a power analysis was not performed. We present 95% CI of estimates to indicate the range of effect sizes supported by the data.

## Results

**Fig. 1:**
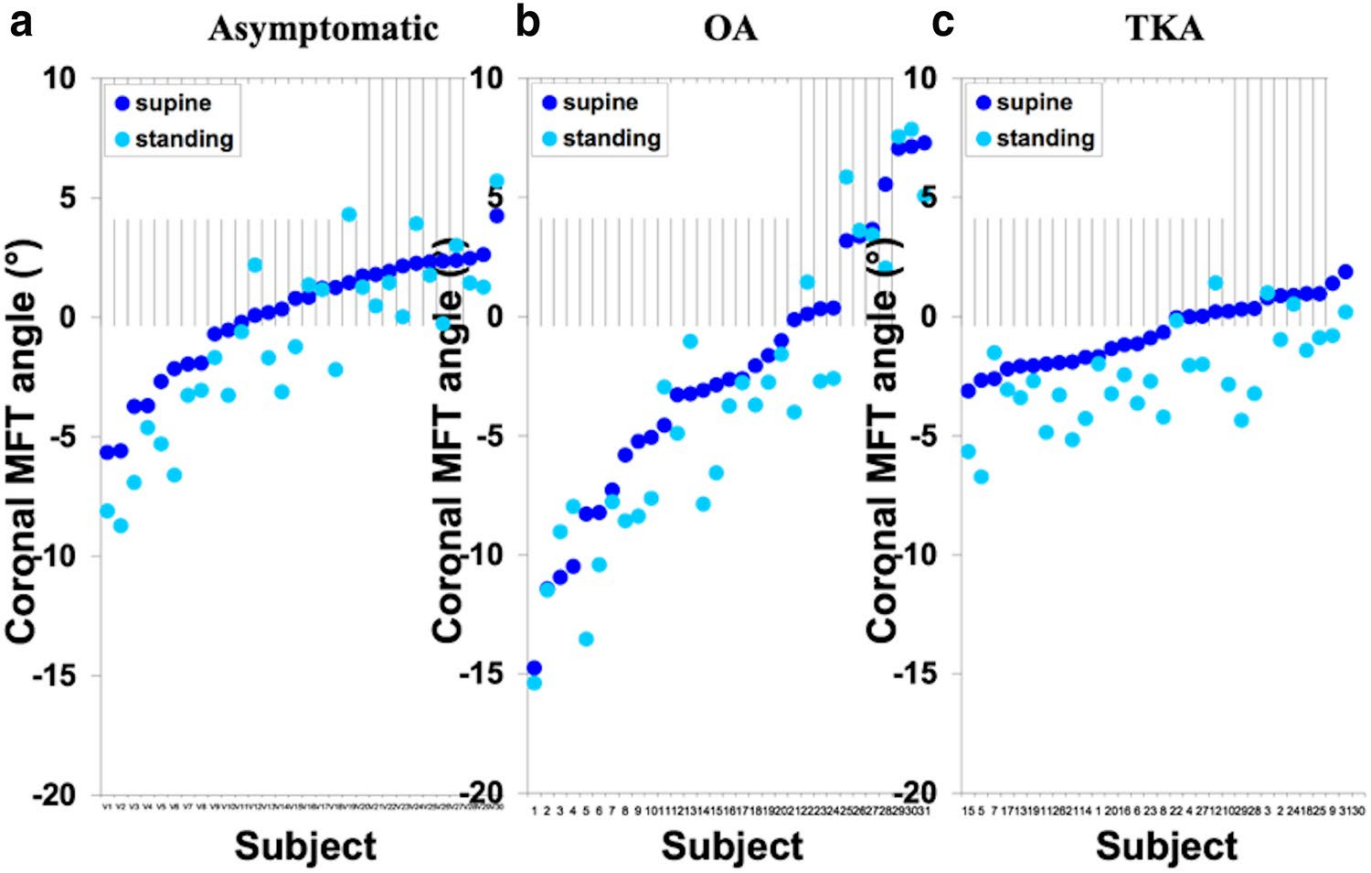
Coronal MFT angles (°) supine (dark blue) and standing (light blue) for all subjects in each group. Subjects have been ordered based on supine alignment

**Fig. 2:**
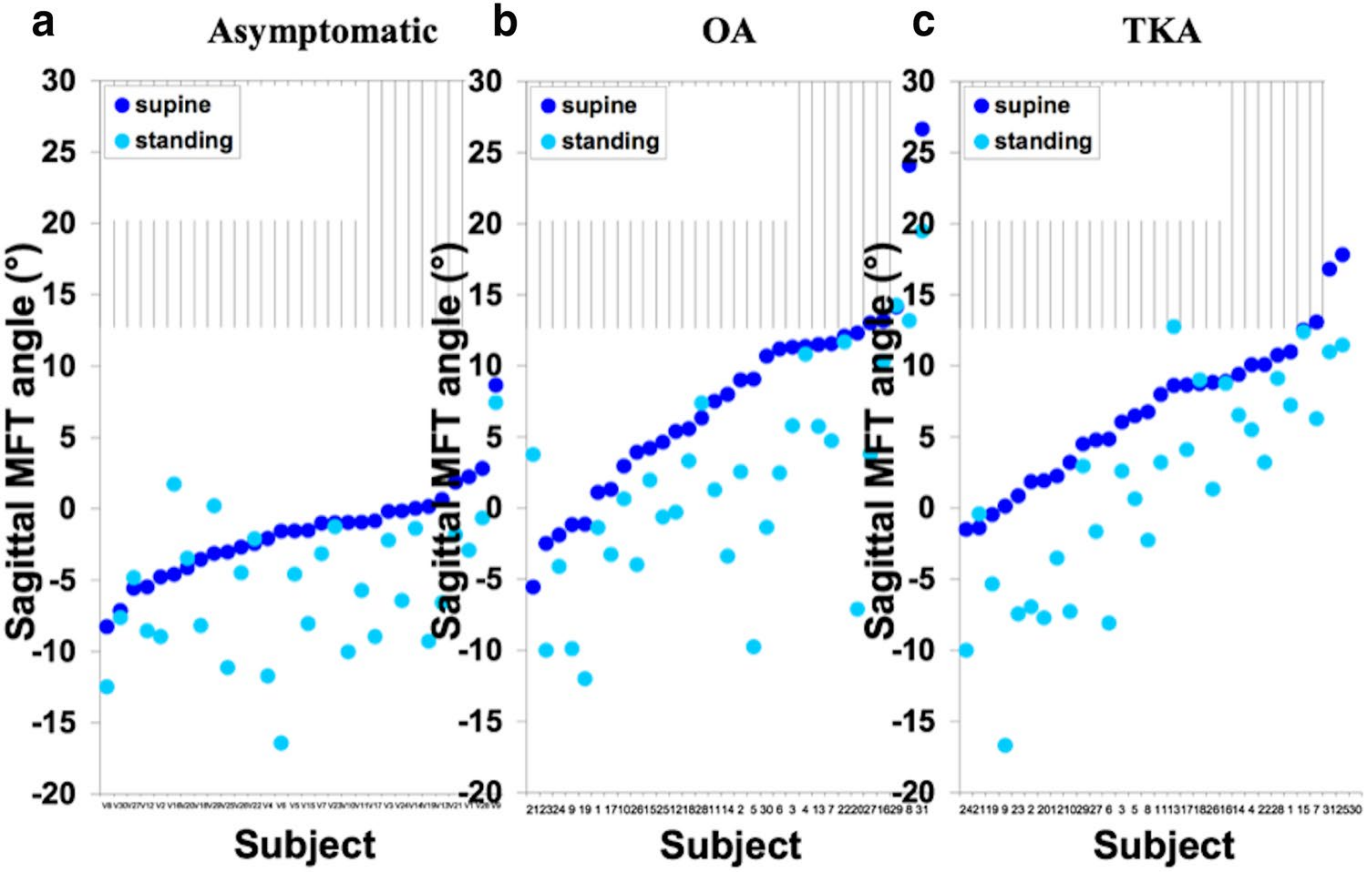
Sagittal MFT angles (°) supine (dark blue) and standing (light blue) for all subjects in each group. Subjects have been ordered based on supine alignment

**Fig. 3:**
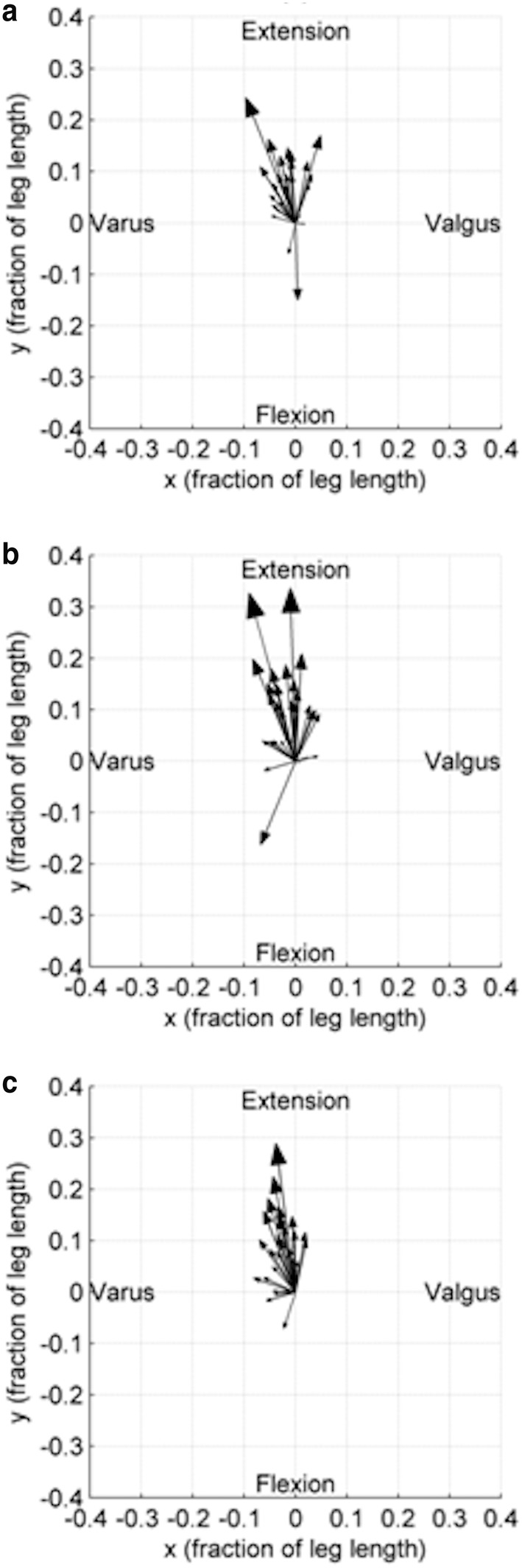
Relative ankle centre displacement with respect to knee centre from supine to standing. **a** Asymptomatic, **b** OA and **c** TKA knees. Arrowhead size denotes magnitude of change. For these plots flexion is negative and extension is positive

The asymptomatic group consisted of 19 males and 11 females; the mean age was 41 years (20–65) and mean BMI was 26 kg/m^2^ (19–34). For the OA group there were 18 males and 13 females, mean age was 66 years (52–82) and mean BMI was 33 (23–43). The TKA group was the same apart from one female was excluded. The mean and range of coronal and sagittal MFT angles for each group are given in Table 1. All three groups showed a trend towards varus and extension when going from supine to standing (Figs. 1, 2). The asymptomatic group showed a mean change of − 1.2° (95% CIs − 1.8, − 0.5), *p* = 0.001 in the coronal plane and − 3.8° (95% CIs − 5.4, − 2.2), *p* < 0.001 in the sagittal plane. For the OA group this was − 1.1° (95% CIs − 1.9, − 0.3), *p* = 0.009 and − 5.9° (95% CIs − 8.0, − 3.9), *p* < 0.001 respectively. For the TKA group in the coronal plane the mean change was − 1.9° (95% CIs − 2.4, − 1.3), *p* < 0.001 and in the sagittal was − 5.6° (95% CIs − 7.3, − 4.0), *p* < 0.001. For the three groups, the direction and magnitude of the displacement vector plots show the combined trend of relative varus and extension for most subjects (Fig. 3).

**Table 1:** Coronal and sagittal MFT angles for each group

MFT angle	Asymptomatic	OA	TKA
Coronal			
Supine	0.1 (2.5) [− 5.7 to 4.2]	− 2.5 (5.7) [− 14.7 to 7.3]	− 0.7 (1.4) [− 3.1 to 1.9]
Standing	− 1.1 (3.7) [-8.7 to 5.7]	− 3.6 (6.0) [− 15.4 to 7.8]	− 2.5 (2.0) [− 6.7 to 1.4]
Change	− 1.2 (*p* = 0.001)	− 1.1 (*p* = 0.009)	− 1.9 (*p* < 0.001)
Sagittal			
Supine	− 1.7 (3.3) [− 8.3 to 8.7]	7.7 (7.1) [− 5.6 to 26.6]	6.8 (5.1) [− 1.5 to 17.8]
Standing	− 5.5 (4.9) [− 16.4 to 7.4]	1.8 (7.7) [− 12.0 to 19.5]	1.4 (7.6) [− 16.7 to 12.7]
Change	− 3.8 (p < 0.001)	− 5.9 (*p* < 0.001)	− 5.6 (*p* < 0.001)

Data are presented as mean (SD)[range] with varus alignment/hyperextension being negative and valgus/flexion being positive

## Discussion

The most important finding of the present study was that change in MFT angle from a supine position to bipedal stance was to more relative varus and extension, regardless of the type of knee or the initial alignment. This finding suggests that the soft tissue restraints may be more important that the rigid bony or prosthetic architecture for controlling this weight-bearing alignment change.

The knee is a load-bearing joint and its alignment is therefore normally assessed in the standing position. However surgical interventions such as TKA are performed on supine limbs with the components placed in the desired target position using either traditional or computer-assisted guidance systems. Limbs are then re-evaluated radiographically in the standing position which serves as a measure of whether the desired intra-operative alignment targets have been achieved. Potential differences between anaesthetized and awake patients along with radiographic measurement errors, have meant that the relationship between supine and standing knee alignment is poorly understood [12].

Our findings were support by those from a previous radiographic study in which it was showed that coronal MFT angle in varus OA knees was more varus on standing than supine [25]. Another study, using Computed Tomography (CT) to assess changes in the patellofemoral joint alignment between supine and standing, found that the medial joint space narrowed upon standing which would support our findings of a more varus alignment upon standing [13]. Other studies have shown changes in coronal alignment between supine and standing positions using different imaging modalities to assess alignment in each position [9, 22, 28]. Although Shoenmakers et al. reported a difference, the way their results are presented (focusing on agreement between the two modalities) means it is not possible to know whether their TKA knees were more varus on standing [22]. Gebjuade et al. also reported a difference while focusing on agreement between modalities but their overall results did not show that knees became more varus on standing [9]. However, their cohort contain a high proportion of valgus knees and the detailed results appeared to show varus knees having to tendency to more varus on standing and valgus knees having a tendency to more valgus [9]. Similar results to this were found by other authors [11, 27, 28]. Winter et al. showed that in OA (pre-operative) knees measured deformity increased on standing, with in general varus and neutral knees becoming more varus which supports our findings [28]. However, the use of different imaging modalities to measure alignment could represent a significant limitation [9, 11, 22, 27, 28]. Meijer et al. found that both before and after prosthesis implantation that knees were more varus on standing, which supports our results, although there was some evidence that high valgus (pre-operative) knees became more valgus [18]. Recent work using the same non-invasive system to measure the MFT angle for 264 knees in 132 healthy volunteers found that when the lower limb was initially in 2.5° valgus or less it tended to become more varus and hyper-extended upon standing [7]. The authors concluded that the consistent kinematic pattern observed suggested that soft tissue constraints, rather than underlying joint deformity are more influential than control of alignment from lying to standing. This supports our findings but we have also shown that this pattern holds true for osteoarthritic and prosthetic knees which may have important clinical implications.

The relative change to varus observed is another important consideration when performing TKA, on supine patients with a target MFT angle window. Traditionally this MFT angle was widely accepted as 0 ± 3° with concerns that outliers could limit implant survival and decrease patient satisfaction [20]. Subsequent work has brought the validity of this target into question, with more recent studies showing no clear relationship between malalignment and decreased implant survival [4, 19]. Other studies have shown that patients do well in terms of function and survivorship following TKA when left with some residual varus as opposed to correcting to a neutral MFT angle target [17, 26]. These findings have led to the suggestion that individual alignment targets may be more appropriate since outcomes did not correlate well with alignment [23]. A cadaveric study showed that positioning implants in the patient’s “constitutional position” most closely replicated the pre-operative ligament tensioning [8].

Although there is no clear consensus on how bony cuts and target alignment affect patient satisfaction and implant survivorship following TKA, it is clear that one target does not provide the perfect knee for all patients. Our finding of consistent trend to varus and extension upon standing regardless of the initial alignment is likely to have important implications for arthroplasty surgeons. If we consider that the soft tissue restraints are likely to be more important than previously thought for determining post-operative weight-bearing alignment then we must quantify this soft tissue behaviour before undertaking TKA.

There are only a few studies addressing the changes in sagittal alignment from supine to standing. Our results showed from lying to standing there was a statistically significant trend to relative extension ranging from a mean of 4° for healthy knees to approximately 6° for both the OA and TKA groups. The magnitude of the difference appeared to be unrelated to supine maximum extension angle. This trend to relative extension may suggest a natural response to attempt to “lock” the knee during prolonged standing. From a flexed position, a posterior translation of the knee joint centre with regards to the body centre of mass, will reduce the required knee extension moment. This implies less energy required for active muscular contraction and reduced contact stresses in the knee. Much of the previous work addressing flexion deformity following TKA has assessed sagittal alignment using a goniometer in the supine position. This may be due to difficulties associated with obtaining full length weight-bearing lateral radiographs [24]. The predictable trends to relative extension upon standing shown in our results suggest that supine assessment of sagittal alignment is likely to be misleading. We would therefore conclude that patients should also be assessed in a standing position. Prosthetic knees with post-operative sagittal alignment of between 5° flexion and 9° hyperextension have been shown to have better outcome scores than those which lie outside this range [20]. The results of this study may offer some explanation for these findings. Patients with small flexion deformities would be likely to correct upon standing. Conversely, intra-operative hyperextension due to excessive bony cuts or soft tissue releases may be further exacerbated during weight-bearing. With respect to intra-operative TKA alignment targets in the presence of fixed flexion deformities, these results suggest it may not always be necessary to achieve complete correction of a fixed flexion in order to achieve a sagittal MFT angle of 0° when standing although further work in this area with a larger sample size is required.

There are limitations to this study. Our patient numbers in each cohort are relatively small; however all of our key findings were statistically significant. Our cohort had a range of alignments but there were no patients who had significant valgus alignment therefore the trends we have observed may not hold true for patients with large valgus knees. This paper reports a secondary outcome of another study [5]. As the study was not powered on this outcome, a power analysis is not presented. As per best practice we report 95%CI of estimates to inform the reader as to the likelihood of an inadequate sample size [16] rather than carrying out a post-hoc power analysis which has been shown be to inaccurate and misleading [10]. A further limitation is that we cannot assess the effect of demographics on changes in alignment as the study was not set up for this, The asymptomatic group were younger than the OA/TKA groups, however as the results are consistent across the three groups the variation in demographics between groups does not appear to have a large influence.

## Conclusion

In conclusion, this study has observed consistent changes in MFT angles between supine and standing positions across all knee types. We can therefore reject our hypothesis that we expected osteoarthritic and prosthetic knees to show different trends in alignment changes to asymptomatic knees. The relative displacement to extension and varus upon standing suggests that changes to the joint contact surfaces, either through osteoarthritic changes or TKA, only have a small effect on dynamic alignment changes when going from a non-weight-bearing to a weight-bearing situation. This leads to the conclusion that the soft tissues play an important role in this dynamic behaviour and highlights the importance of quantifying soft tissue behaviour when planning, performing and evaluating alignment dependent surgical interventions of the knee. When routinely assessing any type of knee, clinicians should be aware that subtle consistent alignment changes occur under weight-bearing conditions and tailor their treatments accordingly.
